# Insights from the judgement bias paradigm: social group and tank size do not affect mental state in female guppies

**DOI:** 10.1111/jfb.15481

**Published:** 2023-06-29

**Authors:** Judith Epping, Alexander Kotrschal, Séverine D. Kotrschal

**Affiliations:** ^1^ Department of Animal Sciences, Behavioural Ecology Wageningen University Wageningen The Netherlands

**Keywords:** guppies, housing conditions, judgement bias test, mental state, positive welfare, resilience

## Abstract

Optimal holding conditions are key to animal welfare. How stressful husbandry is perceived by the animal can be determined via an assessment of an animal's mental state – where it is positioned on the continuum between optimistic and pessimistic states – and can be measured using the judgement bias paradigm. In this test, individuals are trained to distinguish a rewarded from an unrewarded cue before being presented an ambiguous, intermediate cue. The response time to the ambiguous cue is then indicative of mental state. A shorter latency suggests a more positive (optimistic) mental state and a longer latency a more negative (pessimistic) mental state. Here, the authors used the judgement bias paradigm to assess the impact of standard laboratory housing conditions on the mental states of female guppies (*Poecilia reticulata*). As it is debated which holding conditions confer optimal welfare, they tested the impact of husbandry on mental state by keeping animals for 3 weeks in small or large social groups in either small or large tanks. They found that the different standard housing conditions used did not lead to differences in mental state. As an unexpected side result, they found that female guppies seem lateral. Their findings of comparable mental state across housing conditions suggest either that guppies perceive the tested conditions as equally stressful or, alternatively, that guppies are relatively resilient to the combination of group and tank sizes tested in this study. The authors conclude that the judgement bias paradigm can be a useful tool to assess fish welfare.

## 1 INTRODUCTION

The increasing emphasis on animal welfare in science parallels the increasing social awareness of the ethical treatment of animals. An increase in welfare status can be achieved by increasing the resilience of animals (Colditz & Hine, 2016), allowing them to recover more quickly from the negative impact of physical and social stressors and disease challenges (Rutter, 2006). Nonetheless, targeted breeding for higher resilience is often not feasible, for instance, when maintaining the genetic diversity in a population is necessary, such as when breeding animals for reintroduction programmes or basic research. In those cases, optimal holding conditions that control for as many stressors as possible are key to animal welfare. How can one determine which housing conditions are optimal or perceived as the least stressful? As it is not possible to ask animals directly what type of captive environment they prefer, proxies are used, such as low levels of stress hormones (Hoeglund *et al*., 2022), an absence of stereotypical behaviours (Rose *et al*., 2017) or fast growth and high reproductive rate (Sevier *et al*., 2019). A more holistic approach as to how animals “feel” when exposed to different housing or handling conditions is the judgement bias paradigm test. Judgement bias is the difference in interpretation of and reaction to a stimulus depending on an individual's mental state (Bethell, 2015), whereas mental state is defined as an emotion‐like process controlling certain behavioural and physiological responses and is influenced by internal factors, such as hormones, and external factors, including habitat quality or presence of conspecifics (Douglas *et al*., 2012; Laubu *et al*., 2019; Mendl *et al*., 2009). The more positive experiences an animal made in the past, the more likely it is to be in an optimistic mental state, whereas negative experiences are more likely to lead to a more pessimistic mental state (Laubu *et al*., 2019). In a judgement bias experiment, focal animals are first trained to discriminate between a rewarded and an unrewarded stimulus before being presented with an ambiguous, intermediate cue. The latency, which is the time span between presentation of the ambiguous cue and an animal's response to the ambiguous cue (Bethell, 2015), reflects their mental state: shorter latencies are indicative of a positive mental state or optimistic bias, and longer latencies are indicative of a negative mental state or pessimistic bias (Bethell, 2015). The more affected an animal is by the exposure to different physical and social stressors (here different housing conditions), the larger the latency difference expected to be when tested in a judgement bias test in comparison to control animals that were not exposed to this stressful condition. Or, in turn, the more stable an animal's mental state is when exposed to different housing conditions, the less likely it is that the latter are perceived as significantly more or less stressful, or the more resilient the animal is towards those respective conditions that are perceived as different in terms of stress level exposure.

Experiments for testing mental states using judgement bias tests are widely used in humans and animals (Kruglanski & Ajzen, 1983; Lagisz *et al*., 2020), including bees (Bateson *et al*., 2011), dogs, primates (Mendl *et al*., 2009) and fish (Cerqueira *et al*., 2017; Laubu *et al*., 2019; Rogers *et al*., 2020). In fish these tests primarily focus on social encounters. For example, Laubu *et al*. (2019) could show that female cichlids that were assigned to a non‐preferred partner as assessed in a previous mate choice test exhibited pessimistic bias, which indicates a negative mental state. Similarly, Rogers *et al*. (2020) tested the territorial Murray cod (*Maccullochella peelii*) in a judgement bias test either after being kept with a larger and thus more aggressive conspecific or when having been paired with a smaller and thus less aggressive fish. The authors could show that losing an aggressive encounter seems to result in a less optimistic mental state. Although the judgement bias paradigm seems ideal to test standard husbandry to develop ethical guidelines on positive welfare, only a few such studies have been performed [*e.g*., in rats (Harding *et al*., 2004) and chickens (Zidar *et al*., 2018)]. No such study has been done in fish so far.

Here the authors use the judgement bias paradigm to test how standard holding conditions relating to social group sizes and tank sizes affect the mental state in adult female guppies (*Poecilia reticulata*). Regulations on holding conditions for guppies differ widely and are typically derived from data on other widely used model organisms such as the zebrafish (*Danio rerio*), are based on educated guesses or are lacking entirely. Rare evidence‐based recommendations mainly concern the operational sex ratio, which, if male biased, not only increases male–male aggression but also leads to more coercive mating attempts (Jirotkul, 1999; Kiritome *et al*., 2012). Both these immediate effects of having more males than female guppies in a tank are very likely to be stressful for male and female guppies (Kempkes, 2010). For this experiment the authors tested two laboratory conditions that are standard for keeping females in the laboratory at Wageningen University, the Netherlands. These housing conditions included females being held in small groups of two or six fish and at densities of 1 l water per fish (the minimal density requirement for this and other guppy laboratories visited) or 3 l water per fish. The authors predict that larger group size should induce a more positive mental state than smaller groups, because female guppies naturally live in groups of up to 75 adults (Magurran, 2005). Similarly, the authors expected higher density, *i.e*., less space for each guppy, to induce a positive mental state because guppies are shoaling fish and closer contact to a group decreases the (perceived) risk of predation (Lachlan *et al*., 1998). They did not expect intraspecific competition for resources to negatively affect mental states with higher density (Bassar *et al*., 2013), as fish were fed to saturation.

## 2 MATERIALS AND METHODS

### 2.1 Experimental set‐up and experimental apparatus

To test for a judgement bias (*i.e*., differences in the mental state) of guppies kept under different standard laboratory housing conditions, the authors performed an experiment in three phases: an initial pretraining during which fish had to learn how to dislodge a plastic disc to find food underneath and a spatial discrimination learning task (phase 1: 2 weeks each), a 3 week exposure to four different housing conditions with varying group sizes and density regimes (phase 2) and a final judgement bias test during which the authors measured the latency time of the focal fish to approach an ambiguous cue, a disc positioned exactly in the middle of the beforehand rewarded and unrewarded discs (phase 3).

**FIGURE 1:**
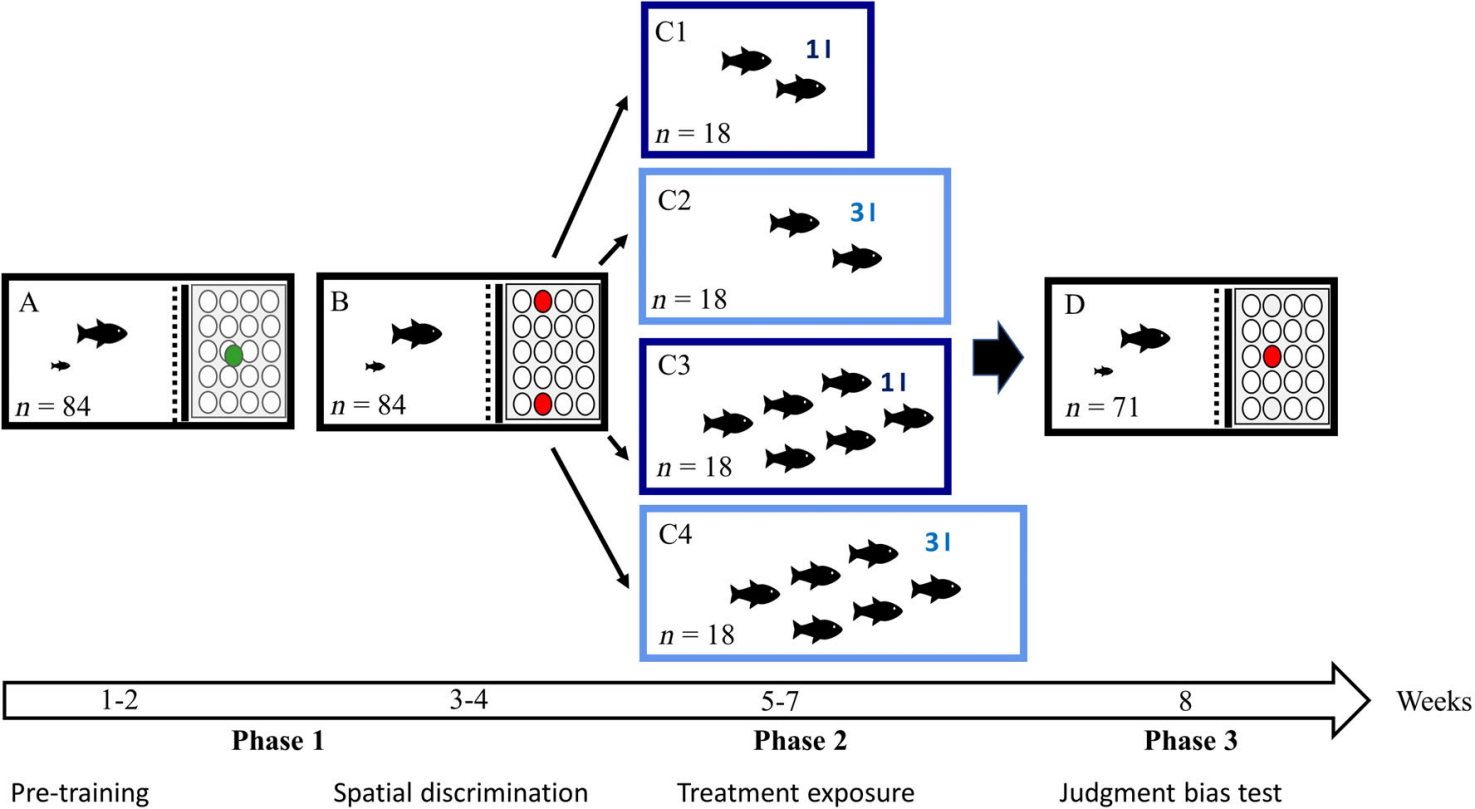
The experimental set‐up and timeline of the study. Depicted is the set‐up of the learning tanks (A, B, D) each consisting of a housing compartment (left, image with focal female and companion) and an experimental compartment (right, image with training plate) separated by two sliding doors (solid and dashed lines in between these two compartments) and the set‐up of the four treatment tanks (C1–C4) with sizes 2, 6,6 and 18 l, reflected by the relative size of tanks in the figure. During the first 4 weeks (phase 1) of (A) pretraining and (B) spatial discrimination learning, female guppies learnt to associate a food reward hidden under a red disc on either the left or right side of the plate> For the following 3 weeks (phase 2), all females got exposed to one of four different housing conditions (C1–C4), which differed in group size (two *vs*. six fish, as indicated by black fish symbols) and fish density [1 l of water per fish (dark‐blue rectangles) *vs*. 3 l of water per fish (light‐blue rectangles)], before their performance in a judgement bias test (D) was assessed in phase 3 of the study

All guppies used in this experiment are descendants of wild fish caught in the upper Aripo River, Trinidad, and have been kept in the laboratory for more than 10 generations. The authors used female‐only groups as females form stronger social bonds in the absence of male individuals (Darden & Watts, 2012), and male harassment via coercive mating attempts likely adversely affects mental state (Dadda, 2015) and was therefore excluded from this study. Focal females (*n* = 84) were kept in learning tanks (40 × 15 × 15 cm) throughout the initial pretraining and spatial discrimination learning phase (phase 1) and were returned to the tanks (after a 3 week period of exposure to different housing conditions) for the judgement bias test (phase 3; see Figure 1). Learning tanks included a housing chamber (15 × 15 × 15 cm) and an experimental chamber (25 × 15 × 15 cm). Both chambers were separated by an opaque sliding door and a transparent sliding door. The experimental chamber included a white plate with 20 holes in which frozen food rewards (*Artemia*) were placed and covered with plastic discs that the fish could either dislodge as learned during the pretraining phase (rewarded stimulus) or that were immovable for the fish by means of a glued‐on silicon knob (unrewarded stimulus). The experimental timeline and the learning apparatus are shown in Figure 1 (also see Buechel *et al*., 2018), and this learning set‐up has been successfully implemented in cognition studies with female guppies in the past (*e.g*., Boussard *et al*., 2020, Buechel *et al*., 2018, Fong *et al*., 2019). Each focal female was housed with one juvenile companion, and the housing chamber had visual contact with the surrounding tanks to avoid social isolation effects (Brandão *et al*., 2015). All guppies were acclimatised to the learning tanks for 2 days. During acclimatisation the females had access to both the housing and experimental chambers, and the white plate that was later used in the experiment could be explored by the fish to reduce the effect of novelty objects in the trials (Brown & Laland, 2002). Tank standard conditions, for stock tanks, learning tanks and treatment tanks, included a gravel layer and java moss (*Taxiphyllum barbieri*), with constant aeration ensured by a flow‐through system with a biological filter and weekly water changes (for details on water measurements, see Supporting Information Table S1). The mean water temperature was 24 ± 2°C with a 12 h dark–light regime. Fish were fed with frozen *Artemia* during trials (phases 1 and 3) and once a day with flake food until visual satiation during the housing treatment (phase 2).

### 2.2 Experimental procedure, phase 1: pretraining and spatial discrimination learning

During the pretraining (see Figure 1, phase 1, A), females had to learn to dislodge a plastic disc on a plate with holes to find a food reward underneath. The full procedure is described in Buechel *et al*. (2018). In brief, frozen adult *Artemia* were placed in one of the holes of the experimental plate, with a green disc placed next to the hole. The green disc was moved to stepwise cover the entire hole in consequent trials until the disc was entirely covering the hole such that the focal animal needed to dislodge the disc to find the reward underneath. This task imitates the natural foraging behaviour of wild guppies that search for food under leaves (Houde, 2019). Each female needed to successfully dislodge the disc thrice in a row to take part in the following spatial discrimination learning task. For spatial discrimination learning (Figure 1, phase 1), two holes were covered with red discs, one on the left and one on the right. One disc could be dislodged by the fish, and the other disc was fixed in the hole with a glued‐on silicon knob. Rewards were placed underneath both discs to control for olfactory cues. Half of all females (*n* = 42) were trained on the left disc being rewarded, the other half (*n* = 42) on the right disc. During the trials, the opaque sliding door that controlled for the fish being affected by the experimenter's manipulation was opened first, followed 5 s later by the transparent sliding door. This allowed the fish to assess the testing situation and as a result show a more pronounced decision during the subsequent trials. The first attempt to dislodge a disc after the fish entered the experimental chamber was noted as the first correct or incorrect choice, by direct observation of a single experimenter (J.E.). The learning criterion, indicating that the fish had learned the task, was defined as being reached as soon as an individual made at least 80% correct choices over six consecutive trials (*i.e*., five out of six correct choices).

### 2.3 Experimental procedure, phase 2: treatment exposure (housing conditions)

In phase 2 of the experiment, females were assigned to four treatment groups (*n* = 18 focal females, for each treatment group) to be housed in different‐sized tanks and in larger or smaller groups for 3 weeks [see Figure 1, phase 2 (C1–C4) for the different housing conditions]. For treatment C1, females were housed in small tanks (2 l) in pairs at a density of 1 l per fish. In treatment C2, females were housed in pairs in medium‐sized tanks (6 l) at a density of 3 l per fish. In treatment C3, groups of six fish were housed in medium tanks at a density of 1 l per fish. In treatment C4, six fish were placed in large tanks (18 l) at a density of 3 l per fish. To reduce the number of experimental animals, the authors used all females that were trained for and successfully completed the spatial discrimination (*n* = 72) to form the experimental groups. For a sample size of 18 focal females per treatment group in the judgement bias test (phase 3), the authors set up nine tanks (nine replicates) for treatments C1 and C2 (two fish per tank = 9 × 2 = 18 focal females in treatments C1 and C2, respectively; Figure 1, phase 2, C1 and C2) and three tanks (three replicates) for treatments C3 and C4 (with six fish per tank = 3 × 6 = 18 focal females in treatments C3 and C4, respectively, see Figure 1, phase 2, C3 and C4). The authors further grouped females trained on the same side in treatment tanks, but they had four tanks with females trained on both sides (mixed‐side tanks). See Supporting Information Table S2 for an overview of replicates per treatment and side trained on per replicate. After 3 weeks of treatment exposure and before transferring fish back to the learning tanks for the judgement bias test, the authors obtained images of each female for individual identification based on their unique black head pigmentation. This was done to keep track of the respective side each female in the four mixed‐side tanks was initially trained on.

### 2.4 Experimental procedure, phase 3: judgement bias test

Before running the judgement bias test, females were acclimatised to the learning tanks for 2 days and were given three reminder trials, with the food reward hidden on the previously rewarded side during the spatial discrimination learning of phase 1. The authors did this to avoid variation in memory performance affecting the latency measures in the subsequent judgement bias trial. After successfully completing the reminder trials, the focal females (*n* = 71, one jumped out of the tank during acclimatisation) were presented with the ambiguous cue, a red disc placed in between the previously trained rewarded and unrewarded spatial locations during phase 1 of the experiment. The authors recorded the latency (time) of the focal females to attempt to dislodge the disc with ambiguous information. Females were given a maximum time of 90 s to approach and attempt to open the disc before being excluded from the subsequent data analysis. Note that focal females were distinguished only by running numbers to ensure that the experimenter was blind with regard to the treatment.

### 2.5 Data analysis

#### 2.5.1 Spatial discrimination learning

The author performed and analysed the spatial discrimination trials to ensure that all females had learned to associate the food reward with a spatial location (a prerequisite for the judgement bias test). They therefore manually checked whether each individual female had reached their predefined learning criterion of at least 80% correct choices over 6 consecutive trials after 20 spatial learning trials, additionally training those females that had not reached the criterion yet until the criterion was reached. Following the procedure described in Buechel *et al*. (2018), they further ran a generalised linear mixed model (GLMM) to compare the learning rate, number of errors made and the end‐performance level of females trained on the left or right side from a fish perspective. This analysis was done to test for potential side bias in females which could result in one group (left or right side trained on, which is not counterbalanced across treatments in the authors’ design; see “Materials and methods” section and Supporting Information Table S1) making more mistakes while learning the spatial task which in turn could affect their level of optimism in the subsequent judgement bias test (*e.g*., poor learners making more mistakes in a learning task being less optimistic than an individual that learns a task easily making fewer mistakes while doing so). The model to test for a side bias used individual “success” per trial as a binary dependent variable, counted as the first correct or wrong choice. The trained‐on side (left *vs*. right side rewarded), trial (1–20) and their interaction were included as fixed effects and individual learning curves as random effects controlling for repeated measurements per fish [R syntax for the full model: success ~ side * trial + (trial|fish); final model: success ~ side + trial + (trial|fish)]. The authors ran a similar model to compare the respective end‐performance level (proficiency) over the last six trials of females trained on either side [R syntax full and final model: success ~ side + trial + (trial|fish)].

#### 2.5.2 Judgement bias test

To assess the mental state of female guppies under four different housing conditions the authors analysed the latency to attempt to open the ambiguous cue using a linear mixed model (LMM) that included latency time as response variable, the density treatment (1 *vs*. 3 l per fish) and the social treatment (one *vs*. five companion females) as well as the side the focal females were trained on (left *vs*. right side rewarded) and their interaction as fixed effects and replicate as random effect [R syntax full model: latency ~ density * social * side + (1|replicate), final model: latency ~ treatment + side + (1|replicate) and final model: latency ~ density + social + trial + (1|replicate)]. To fulfil the normality requirement, the authors power‐transformed the latency time data (transformation parameter = 0.237) using the car package in R (Fox & Weisberg, 2018). They further ran a linear regression model (LM) to test for a potential correlation between mean individual success in the spatial discrimination task and the respective judgement bias response (R syntax: success ~ latency). They ran this model to test the idea that less efficient learners that make more errors while learning a task are less optimistic with longer latencies to approach an ambiguous cue in the judgement bias paradigm. All analysis was performed in R statistical software version 4.0.5 (R Core Team, 2021) using the lme4 package for linear mixed‐effects modelling in R (Bates *et al*., 2015). Final models were determined using stepwise elimination of non‐significant interaction terms and AIC.

## 3 RESULTS

### 3.1 Spatial discrimination learning

Seventy‐two out of the initial 84 females had learned to associate a spatial location with a food reward based on the authors’ predefined individual learning criterion [mean 12 ± 4 (s.d.) trials until the learning criterion, five out of six consecutive trials, was reached]. For the analysis of side bias in the focal females, the authors included only the data collected in trials 4–20 in the model, as in the first three trials only one‐third of fish made a choice for either disc during the trials. Between trials 3 and 20 however, both groups (left and right side rewarded) learnt to associate the side with the food reward (GLMM; trial: *z* = 3.189, *P* = 0.001), and there was no difference in learning rate (slope of the learning curve) between fish trained on the different sides (right *vs*. left side rewarded) (GLMM; side * trial: *z* = −1.176, *P* = 0.239). Females trained to find the reward on their left side (from the fish perspective) however made fewer mistakes while learning the spatial task than females trained to find the reward on their right side (GLMM; side: *z* = 2.470, *P* = 0.014). The groups achieved a comparable end‐performance level of 95 ± 1% (right side) and 97 ± 1% (left side) over the last six trials (GLMM; side: *z* = 0.611, *P* = 0.541).

### 3.2 Judgement bias test

**FIGURE 2:**
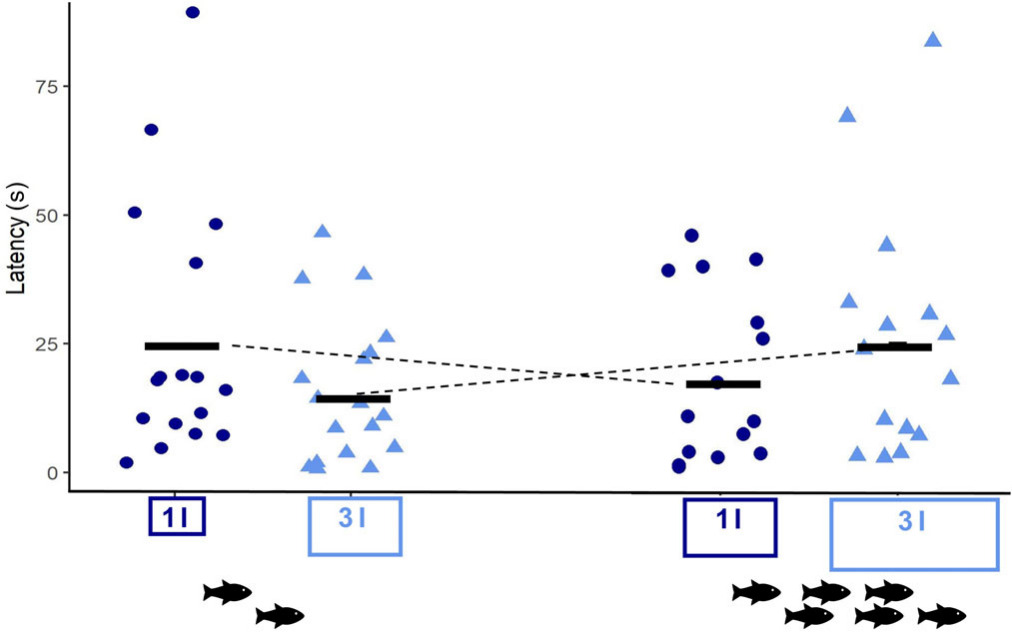
Judgement bias test results showing no significant difference in the latency of female guppies (*n* = 71) to attempt to open a disc in an ambiguous position (with reference to a rewarded and non‐rewarded position trained on in a spatial discrimination task) after a 3 week exposure to four different housing conditions consisting of two different group sizes (two *vs*. six fish, as indicated by black fish symbols) and two different density regimes [1 l of water per fish (dark‐blue rectangles) *vs*. 3 l of water per fish (light‐blue rectangles)]. Dark‐blue dots represent the raw data for the high‐density treatments with 1 l of water per fish, and light‐blue triangles show the raw data for the low‐density treatments with 3 l of water per fish; group means are indicated by black solid lines. Dashed lines show a potential trend for a between‐treatment group interaction [linear mixed model (LMM): *F* = 2.979, *P* = 0.08]

Analysis of latency to attempt to open the ambiguous cue in the judgement bias test revealed substantial variation (range: 0.69 ± 19.3 to 89.47 ± 19.3 s) but no significant differences between the four different housing conditions (LMM; density: *F* = 0.169, *P* = 0.681; social: *F* = 0.123, *P* = 0.727; Figure 2). Note, however, that there was a non‐significant interaction trend towards females kept in smaller groups having a shorter latency time in the judgement bias test when kept at lower density (3 l per fish), whereas females kept in larger groups tended to prefer less space per fish (1 l per fish) (LMM; density * social: *F* = 2.978, *P* = 0.08; Figure 2). The sides females were trained on did not significantly affect response times in the judgement bias task (GLM; side: *F* = 0.373, *P* = 0.55), and there was no significant relationship between individual mean success in spatial discrimination learning and judgement bias latency (LM; latency: *t* = −1.214, *P* = 0.229).

## 4 DISCUSSION

The authors trained female guppies in a spatial associative learning task to assess their mental state in a judgement bias paradigm after being housed under different standard laboratory conditions. Their aim was to reveal potential preferences for different housing conditions (four different treatments, two different levels of social interaction and space use), and they expected group size to be the strongest predictor for the mental state of adult female guppies because of the social bonds the latter form in natural groups (Darden & Watts, 2012). Although the authors found that female guppies learn the spatial task readily and make more correct choices when trained on the left side compared to females trained to find the food reward on their right side, they did not find significant latency differences between female guppies exposed to different housing conditions but a non‐significant trend towards an interaction between group size and density preferences. They interpret the result as guppies either perceiving the tested conditions as equally stressful or, alternatively, being relatively resilient towards variation in standard housing conditions.

The authors use the term “relatively resilient” for two reasons. The first is because the weak interaction trend between group size and density preferences might indicate that under given circumstances the holding conditions indeed do affect the mental state of female guppies, and the second is because their focus was on testing the animals in a positive welfare set‐up under standard laboratory conditions. Therefore, the authors abstained from exposing animals to housing conditions that are thought to be stressful for guppies as, *e.g*., evoked by housing guppies in social isolation (Magurran, 2005) or by keeping females with many males (Magurran & Ojanguren, 2007). They used this presented experimental design, even though they were aware that using more stressful treatments (a negative reference condition) may potentially have revealed differences in mental state, highlighting that for female guppies it does indeed matter how they are kept, which in turn would have important consequences from a welfare perspective. This being said, it is also important to mention that the authors’ experimental design does not allow for drawing conclusions as to whether female guppies kept under these specific conditions tested here generally are in an optimistic mental state or generally in a more pessimistic mental state. For example, it is possible that living in such unnatural small groups of two or six females *per se* is perceived as stressful for guppies that tend to form loose shoals with up to 75 individuals (Magurran, 2005). Further research in this direction is needed to understand the impact of holding conditions on stress levels, inducing a pessimistic mental state in guppies or fish in general.

Besides manipulating group size, the authors tested the effect of two different densities in the tanks. They expected higher density (more fish per litre of water) to induce a more positive mental state when compared to guppies kept at lower densities. This expectation is based on a lowered individually perceived risk of predation and inherent shoaling preferences (Lachlan *et al*., 1998) with less use of space. Their results, however, did not support this idea. Exposure to the two different density treatments used in this study did not affect the mental state of female guppies. One reason that may explain their result is that the fish did not experience any predator‐induced stress. The fact that the fish were fed to visual saturation may also have further masked a potentially negative effect of competition for food. Nonetheless, if fish were exposed to predation in the wild or have limited access to feed in a densely populated tank, density might well play a bigger role in influencing mental states and welfare of fish. Also, handling (netting) of fish may have masked a potential treatment effect on the mental state of female guppies in this study. The authors transferred the females from their treatment tanks back into the learning tanks using nets, which, despite being done by a well‐trained researcher, almost certainly is a stressful experience for the fish (Gibson *et al*., 2009) and may have stronger effects on the mental state than the different housing conditions. A logical next step could thus be to assess the mental state of guppies in relation to the amount of handling time or events. One open question is whether 3 weeks of exposure to the experimental treatment conditions was sufficient to induce differences in mental state. This cannot be currently answered as the duration and persistency of mental states are currently unresolved. Nonetheless, data from female convict cichlids (*Amatitlania siquia*) (Laubu *et al*., 2019) suggest that 3 weeks of exposure should have been enough time. Cichlids were subjected to a series of three judgement bias tests and reminder trials on three consecutive days, which led to significant differences in mental state in females paired with their preferred *vs*. non‐preferred partner in the third judgement bias trial. Differences were thus induced by a mere 24 h period between trials. This supports that guppies are relatively resilient to the housing conditions applied here, allowing them to adapt to the treatment conditions in the 3‐week treatment period.

To run the judgement bias test, the authors pretrained female guppies in a spatial discrimination task. Analysing this data revealed a side bias, which may suggest that the female guppies are lateral. Side bias (lateralisation) is controversially discussed in cognitive research. The expression, or detection, of laterality seems strongly dependent on context and stimulus (Penry‐Williams *et al*., 2022), and repeatability of results often is not given (Roche *et al*., 2020). Nonetheless, the fact that guppies in this study made more errors when trained on one side than the other requires additional analysis. The authors hypothesised that the number of errors and thus negative experiences female guppies have during the process of learning an association can have a negative impact on the mental state of the learner, which might override the experience during the treatment effects that the females were exposed to. The authors’ data, however, did not support this idea. There was no significant correlation between the mean number of errors a female made during learning and the respective latency during the judgement bias test.

In summary, this study being the first of this kind highlights that choosing one over the other tested standard laboratory condition to keep female guppies under stress does not affect the mental state of the fish. As the learning set‐up used here has been successfully adapted for different fish species (cichlids *A. siquia*, Laubu *et al*., 2019, and rainbow trout *Oncorhynchus mykiss*, Anderson *et al*., 2022), there seems to be great potential for this approach in gaining a better understanding of factors affecting the welfare of fish kept in laboratory or private settings.

## AUTHOR CONTRIBUTIONS

S.D.K., A.K. and J.E. designed the study. J.E. collected data. J.E. and SDK performed the data analysis and wrote the first draft. All authors contributed to finalising the paper.

## ETHICS STATEMENT

The care and use of experimental animals complied with animal welfare laws, guidelines and policies as approved by the Animal Welfare Body (Instantie voor Dierenwelzijn, IvD) at Wageningen University and Research. This study was based on behavioural observations, and stress to the animals was minimised as much as possible.

## Supporting information


**TABLE S1.** Water measurements for Epping *et al*. (2023)

**TABLE S2.** Treatment overview

## Data Availability

All data will be deposited on Dryad.
