# Supplementary material for: Insights from the judgement bias paradigm: social group and tank size do not affect mental state in female guppies
Source: J Fish Biol. 2023 Jun 29;106(1):12–9. doi: 10.1111/jfb.15481 (PMC11758249; doi:10.1111/jfb.15481)
Supplement: Supplementary file 1 — TABLE S1. Water measurements for Epping et al. (2023) [file JFB-106-12-s002.docx]

S1: Water measurements for Epping et al., 2023.

| Date | pH |  | Conductivity  µS/cm | NH_4_^+^ mg/L | NO_2_^-^  mg/L | NO_3_^-^  mg/L |
| --- | --- | --- | --- | --- | --- | --- |
|  |  | Temp. °C |  |  |  |  |
|  |  |  |  |  |  |  |
| 15.09.22 | 8,47 | x | 267 | 0 | 0 | 50 |
| 22.09.22 | 8,33 | x | 298 | 0 | 0 | 30 |
| 26.09.22 | 8,39 | x | 350 | 0 | 0 | 50 |
| 05.10.22 | 8,41 | x | 423 | 0 | 0,025 | 50 |
| 12.10.22 | 8,05 | x | 320 | 0 | 0 | 25 |
| 19.10.22 | 8,16 | x | 252 | 0 | 0 | 25 |
| 27.10.22 | 8.37 | 22 | 226 | 0 | 0 | 10 |
| 03.11.22 | 8,17 | 21,8 | 254 | 0 | 0 | 0 |
| 10.11.22 | 8,26 | x | 232 | 0,025 | 0 | 10 |
| 17.11.22 | 8,38 | 21.6 | 225 | 0 | 0 | 10 |
| 24.11.22 | 8,21 | 22,5 | 228 | 0 | 0,1 | 10 |

S1. x refers to not measured values, 0 refers to values below detection limit. pH and conductivity were measured with a WTW Multi 3630 IDS probe. Ammonia, nitrite and nitrate were routinely measured with respective test kits from the Supelco® MQuant® series.
