# Supplementary material for: Insights from the judgement bias paradigm: social group and tank size do not affect mental state in female guppies
Source: J Fish Biol. 2023 Jun 29;106(1):12–9. doi: 10.1111/jfb.15481 (PMC11758249; doi:10.1111/jfb.15481)
Supplement: Supplementary file 2 — TABLE S2. Treatment overview [file JFB-106-12-s001.docx]

S2: Treatment Overview

| Treatment | Replicate | Group size | Density | Position |
| --- | --- | --- | --- | --- |
| 1: pair, high density | 1 | 2 | 1 L/Fish | L |
| 1: pair, high density | 2 | 2 | 1 L/Fish | R |
| 1: pair, high density | 3 | 2 | 1 L/Fish | L |
| 1: pair, high density | 4 | 2 | 1 L/Fish | R |
| 1: pair, high density | 5 | 2 | 1 L/Fish | L |
| 1: pair, high density | 6 | 2 | 1 L/Fish | R |
| 1: pair, high density | 7 | 2 | 1 L/Fish | L |
| 1: pair, high density | 8 | 2 | 1 L/Fish | R |
| 1: pair, high density | 9 | 2 | 1 L/Fish | Mixed |
| 2: pair, low density | 1 | 2 | 3 L/Fish | L |
| 2: pair, low density | 2 | 2 | 3 L/Fish | R |
| 2: pair, low density | 3 | 2 | 3 L/Fish | L |
| 2: pair, low density | 4 | 2 | 3 L/Fish | R |
| 2: pair, low density | 5 | 2 | 3 L/Fish | L |
| 2: pair, low density | 6 | 2 | 3 L/Fish | R |
| 2: pair, low density | 7 | 2 | 3 L/Fish | L |
| 2: pair, low density | 8 | 2 | 3 L/Fish | R |
| 2: pair, low density | 9 | 2 | 3 L/Fish | Mixed |
| 3: group, high density | 1 | 6 | 1 L/Fish | L |
| 3: group, high density | 2 | 6 | 1 L/Fish | R |
| 3: group, high density | 3 | 6 | 1 L/Fish | Mixed |
| 4: group, low density | 1 | 6 | 3 L/Fish | L |
| 4: group, low density | 2 | 6 | 3 L/Fish | R |
| 4: group, low density | 3 | 6 | 3 L/Fish | Mixed |
